# Supplementary material for: The Prediction of Clinical Mastitis in Dairy Cows Based on Milk Yield, Rumination Time, and Milk Electrical Conductivity Using Machine Learning Algorithms
Source: Animals (Basel). 2024 Jan 28;14(3):427. doi: 10.3390/ani14030427 (PMC10854744; doi:10.3390/ani14030427)
Supplement: Supplementary file 1 [file animals-14-00427-s001.zip › animals-2847029-SI.pdf]

Supplementary Information for

The Prediction of Clinical Mastitis in Dairy Cows Based on Milk Yield, Rumination Time, and Milk Electrical Conductivity Using Machine Learning Algorithms

Table S1. Sources of information (automated monitoring system and rotary milking system) and traits measured using variables at different measurement intervals, summed up to the daily values.

Sensor	Trait	Variable	Measurement interval	Unit
Automatic monitoring system	Activity	Activity	Min/2 h	Min/day
	Rumination	Daily Rumination time	Min/2 h	Min/day
		Rumination deviation per 2 h	Min/2 h	Min/day
		Sum of absolute values of the weighted rumination variation	No./2 h	Min/day
		Rumination at daytime	Min/2 h	Min/day
		Rumination at nighttime	Min/2 h	Min/day
		Ratio of rumination time at daytime to that at nighttime	No./2 h	None
Rotary milking system	Milk yield	Daily Milk yield	Kg/day	Kg/day
	Electrical conductivity of milk	Daily percentage of change of the electrical conductivity of milk	No./milking shift	None
		Standard deviation of the largest change in conductivity over the last three shifts	mS/cm/milking shift	mS/cm
		Peak electrical conductivity of milk	mS/cm/milking shift	mS/cm
